# Reactive Oxygen Species (ROS) and Antioxidants as Immunomodulators in Exercise: Implications for Heme Oxygenase and Bilirubin

**DOI:** 10.3390/antiox11020179

**Published:** 2022-01-18

**Authors:** David Travis Thomas, Nicholas R. DelCimmuto, Kyle D. Flack, David E. Stec, Terry D. Hinds

**Affiliations:** 1 Department of Athletic Training and Clinical Nutrition, College of Health Sciences, University of Kentucky, Lexington, KY 40506, USA; dth225@uky.edu; 2 Department of Neurosciences, University of Toledo College of Medicine, Toledo, OH 43614, USA; Nicholas.Delcimmuto@rockets.utoledo.edu; 3 Department of Dietetics and Human Nutrition, University of Kentucky, Lexington, KY 40506, USA; kyle.flack@uky.edu; 4 Center for Excellence in Cardiovascular-Renal Research, Department of Physiology & Biophysics, University of Mississippi Medical Center, 2500 North State St, Jackson, MS 392161, USA; dstec@umc.edu; 5 Department of Pharmacology and Nutritional Sciences, University of Kentucky, Lexington, KY 40508, USA; 6 Barnstable Brown Diabetes Center, University of Kentucky, Lexington, KY 40508, USA; 7 Markey Cancer Center, University of Kentucky, Lexington, KY 40508, USA

**Keywords:** HO-1, biliverdin reductase, BVRA, PPARα, bilirubin, inflammation, metabolic disease, nutraceuticals, vitamin D, vitamin E, nitrate

## Abstract

Exercise is commonly prescribed as a lifestyle treatment for chronic metabolic diseases as it functions as an insulin sensitizer, cardio-protectant, and essential lifestyle tool for effective weight maintenance. Exercise boosts the production of reactive oxygen species (ROS) and subsequent transient oxidative damage, which also upregulates counterbalancing endogenous antioxidants to protect from ROS-induced damage and inflammation. Exercise elevates heme oxygenase-1 (HO-1) and biliverdin reductase A (BVRA) expression as built-in protective mechanisms, which produce the most potent antioxidant, bilirubin. Together, these mitigate inflammation and adiposity. Moderately raising plasma bilirubin protects in two ways: (1) via its antioxidant capacity to reduce ROS and inflammation, and (2) its newly defined function as a hormone that activates the nuclear receptor transcription factor PPARα. It is now understood that increasing plasma bilirubin can also drive metabolic adaptions, which improve deleterious outcomes of weight gain and obesity, such as inflammation, type II diabetes, and cardiovascular diseases. The main objective of this review is to describe the function of bilirubin as an antioxidant and metabolic hormone and how the HO-1–BVRA–bilirubin–PPARα axis influences inflammation, metabolic function and interacts with exercise to improve outcomes of weight management.

## 1. Introduction

Obesity and ectopic lipid accumulation are key contributing hallmarks of metabolic dysfunction, which is the cornerstone of pathogenesis for most comorbidities [1,2,3]. People with a BMI greater than 30 (>30) have an increased risk for obesity-associated comorbidities that include cardiovascular disease, hypertension, insulin-resistant diabetes, dyslipidemia, and certain cancers [4,5,6,7]. Alterations in lipid metabolism also contribute to ectopic lipid accumulation, exacerbating metabolic disorders, especially when combined with limited physical activity. Understanding and combating metabolic dysfunction is essential for improving clinical outcomes and quality of life. 

Obesity treatment has been challenging, and exercise continues to be the foundation for obesity prevention and treatment. Despite the continued interest in exercise training in obesity, there are still challenges, including patients falling short on meeting exercise recommendations over time and limited effectiveness of exercise as a sole driver for stimulating weight loss [8,9,10]. 

A common theme found in the literature on the healthy athletic population is how different nutrients, hormones, dietary supplements, and other forms of ergogenic aids [referred to herein as “exercise enhancers” (EEs)] can improve exercise training outcomes to enhance athletic performance. There is also emerging evidence that EEs may augment the metabolic benefits of exercise and, in some cases, modulate inflammation. This review provides a brief overview of exercise in preventing metabolic dysfunction along with the potential role of select antioxidants (i.e., bilirubin and others), vitamin D, and nitrates on improving metabolic outcomes associated with exercise. The primary focus will then shift to describing bilirubin’s emerging significance as a potential EE due to its role as a strong antioxidant and metabolic hormone. 

**Figure 1:**
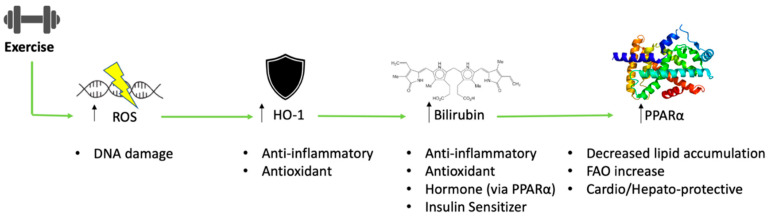
Overview of heme oxygenase and bilirubin interaction with exercise. Exercise increases reactive oxygen species (ROS) and potentiates oxidative DNA damage. The body compensates with oxidative stress by upregulating heme oxygenase-1 (HO-1), which generates the antioxidant bilirubin to help prevent excessive oxidative damage. Bilirubin also directly binds to the PPARα nuclear receptor to induce genes that suppress lipid accumulation and has cardiogenic and hepato-protective effects.

This review describes how exercise interacts with bilirubin to further sensitize these newly defined antioxidant and protective metabolic functions as a hormone. The role of exercise and its influence on bilirubin catabolism will be discussed along with proposed theories on how bilirubin may influence physiological adaptations associated with exercise training and how this might impact inflammatory responses. A primary mechanism discussed postulates that as exercise increases reactive oxygen species (ROS) production, increased heme oxygenase (HO-1) activity raises plasma bilirubin levels, which can also directly bind and activate PPARα (peroxisome proliferator-activated receptor α) in metabolic tissues (e.g., adipose, liver, and muscle), which might explain some of the therapeutic benefits observed with exercise (Figure 1). Other important mediators such as HO-1 and PPARs and their impact on exercise and inflammation will be discussed. 

## 2. The Effect of Exercise on Weight Management and Inflammation

Exercise is regularly prescribed as a first-line treatment in preventing type 2 diabetes, coronary artery disease (CAD), and non-alcoholic fatty liver disease (NAFLD) [11,12,13]. It has strong therapeutic effects that usually meet or exceed expected improvements in metabolic function from pharmaceutical treatment [14]. Exercise training triggers significant metabolic adaptations that improve cardiorespiratory fitness, promoting greater capillary density and increases in HDL synthesis to protect from CAD [14,15]. Exercise also enhances glucose uptake through elevating translocation of GLUT4 in skeletal muscle and by increasing IRS-1 phosphorylation, an insulin receptor substrate that improves insulin sensitivity [16,17]. Therefore, exercise can be a reliable first-line and preventative therapeutic for type II diabetes by decreasing blood glucose [18] and CAD by reducing atherosclerotic plaque buildup and subsequent risk of stroke and myocardial infarction [19].

Although exercise training can improve blood glucose control, insulin sensitivity, and other aspects of metabolic syndrome without weight loss, these benefits are substantially greater when significant weight loss occurs [20,21,22,23]. Indeed, the American College of Sports Medicine issued separate recommendations to maintain health [24] or support weight loss through exercise [25]. Exercise is also one of the primary recommendations of the Diabetes Prevention Program (DPP) and a pivotal component to the classic Look AHEAD trial primarily due to the role that exercise is thought to play in weight loss and weight management [26,27]. Unfortunately, weight loss from exercise is often suboptimal due to compensatory mechanisms that resist the maintenance of an energy deficit [8,9,10]. For instance, an individual may exercise to expend 3000 kcal per week for ten weeks through exercise to expend a total of 30,000 kcal. However, this individual rarely loses 30,000 kcal of body mass. By comparing changes in bodily energy stores with the amount of total energy expended through exercise, we have demonstrated this compensatory response to equate to roughly 1000 kcal per week during a 12-week exercise intervention, and that energy expenditures of greater than 2700 kcal per week are needed to achieve significant weight loss after 12 weeks. [28,29]. Others have reported that greater amounts of exercise can evoke a proportionally greater compensatory response [30], potentially explaining why exercise interventions with large differences in daily and weekly exercise energy expenditures can promote similar weight loss [30,31,32,33]. Because of this, many have turned to various EE’s to improve both weight loss and metabolic health outcomes with exercise. We will discuss some specific studies on EE’s below and how they might impact exercise-induced ROS and inflammation.

### Exercise-Induced Formation of Reactive Oxygen Species and Select Antioxidant Defense Mechanisms 

**Figure 2:**
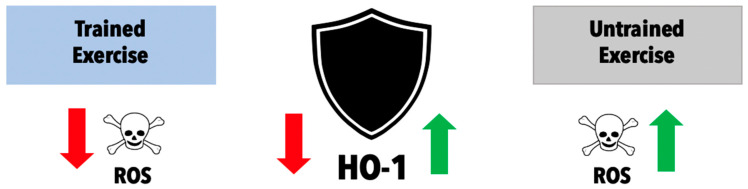
Relationship of HO-1 and ROS in habitually trained versus untrained subjects. Individuals who have performed a single bout of exercise (non-trained) versus individuals with exercise training experience (trained).

**Figure 3:**
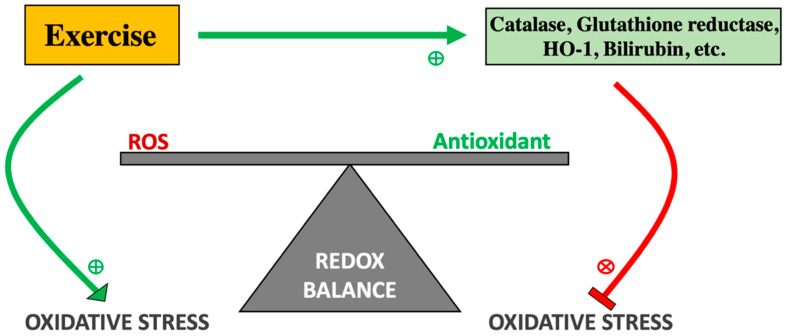
Redox Balance of ROS and Antioxidants. Exercise potentiates the release of reactive oxygen species due to increased oxidative exposure. However, exercise training also induces an adaptive response with the upregulation of antioxidant defense mechanisms that will help restore redox balance. The downregulation of endogenous antioxidant systems or the increased production of reactive oxygen species can precipitate an imbalance in redox balance and potentiate chronic oxidative damage.

ROS generation from exercise has a significant role in triggering and sustaining the healthy cellular, tissue, and organ level adaptations that help improve and maintain cardio-metabolic health. Acute ROS generation from exercise occurs via electron transport oxygen catabolism in the skeletal muscle. This is triggered by a substantial increase in mitochondrial oxygen uptake into the skeletal muscle cell, increasing ROS production [34]. Exercise-induced ROS production can also produce muscle injury, which sends inflammatory signals that attract polymorphonuclear neutrophils and macrophages and produce additional ROS in its defense mechanism of oxidative burst [35]. If there is a chronic imbalance of more ROS production than antioxidant activity, chronic oxidative stress may lead to apoptotic pathways in tissues [36]. A skeletal muscle oxidative stress imbalance is commonly seen in untrained individuals who begin a strenuous training program or “weekend-warriors” who perform a single bout of infrequent strenuous exercise (Figure 2). Although exercise is known to increase the abundance of ROS, progressive exercise training allows time for the upregulation of defense mechanisms that help protect the body from oxidative damage. This is known as redox balance (Figure 3), where free radicals are balanced by the adaptive antioxidants produced [37]. Exercise may stimulate the generation of antioxidants by triggering significant cell adaptations and upregulating antioxidant-producing enzymes [38]. Those who are exercise-trained and perform a single bout of exercise can leverage the benefits of endogenous antioxidant upregulation, along with mitochondrial expansion, cryoprotection, and insulin sensitivity [35]. The importance of ROS is highlighted in experimental models treated with allopurinol. This compound inhibits ROS production and protects muscle tissue from oxidative stress. However, because exercise-induced ROS was not produced, the important adaptive signaling pathways for oxidative protection were blunted. Thus, the formation of ROS in exercise can help activate these intrinsic protective pathways. Notwithstanding, allopurinol is a competitive inhibitor for xanthine oxidase that produces H_2_O_2_. H_2_O_2_ is a kind of ROS; thus, xanthine oxidase is an intrinsic prooxidant. This adaptive signaling response is an important body regulation, and if extrinsic, pharmaceutical dose antioxidants are administered, the body’s natural adaptive ability to produce in trinsic antioxidants may be thwarted [38].

Ultimately, exercise-related ROS adaptations improve oxygen transport and delivery that translate into better aerobic fitness that help explain many of the health benefits of exercise. Furthermore, upregulation of endogenous antioxidant systems can work in concert with exogenous dietary antioxidants to mitigate ROS-related tissue damage and support normal metabolic function and healthy aging. Conversely, the accumulation of ROS and inadequate ROS defense responses has been implicated as a key mechanism leading to significant atrophy in muscle tissue. Muscle atrophy due to chronic excessive ROS exposure progresses slowly as part of the normal aging process but is more pronounced and accelerated in severe underlying pathological processes such as in cancer wasting (cancer cachexia) [39], neurodegenerative diseases (Parkinson’s, Alzheimer’s), and immobilization (musculoskeletal injury) [40]. 

## 3. HO-1, BVRA, and Bilirubin as Inflammatory Mediators

### 3.1. Exercise-Induced HO-1 as a Mediator of Immune System Responses

HO-1 produces a known potent antioxidant and enzyme responsible for the cleavage of heme, yielding biliverdin, iron (Fe^2+^), and carbon monoxide (CO) [41,42]. The HO-1 pathway also regulates some of the metabolic and inflammatory aspects of insulin resistance. While there is a connection between inflammation and the development of insulin resistance, it is unclear which development precedes and which is causative [43]. HO-1’s role in inflammation and insulin resistance appears equivocal in the literature.

The presence of HO-1 mimics the same efficacious properties as bilirubin [44]. Bilirubin downregulates the M1 macrophages associated with the release of pro-inflammatory cytokines [45,46,47]. Future work to understand how HO-1 may affect M1 cells may shed light on potential underlying mechanisms to explain how bilirubin downregulates M1 cells. HO-1 also protects the liver from ischemia-reperfusion injury by modulating the macrophage phenotype into the anti-inflammatory M2 state in mouse livers [48,49]. This serves as evidence for an HO-1 role as a hepatic cryoprotective agent. In this same study, low HO-1 mRNA levels in human liver transplants correlated with increased expression of M1 pro-inflammatory markers [48,49]. Liver-specific biliverdin reductase A (BVRA) knockout animals with reduced hepatic bilirubin had worsened fatty liver on a high-fat diet compared to littermate controls [50], which was confirmed in global BVRA knockout animals [51]. Bilirubin reduces lipid content and inflammatory markers in mouse models of obesity-induced NAFLD [46,47].

Work by Gobert et al. found HO-1 to prevent an inflammatory response and has implicated HO-1 as a virulence factor in *H. Pylori* and other bacteria in order to evade the immune system [52]. Other work has described using a heme-inducing compound to effectively reduce obesity, insulin sensitivity and increase serum adiponectin levels. Inhibition of the HO-1 system decreased adiponectin and increased pro-inflammatory cytokines, TNFα, IL-6, and IL-1 [53,54,55]. Adiponectin, a known anti-inflammatory hormone, is thought to be working indirectly through HO-1-activation [56]. The complete mechanism of this anti-inflammatory activity is not fully understood, but some theories with convincing evidence reveal new insight on HO-1 and the importance of its catabolic products. 

The HO-1 pathway can decrease inflammation by producing biliverdin/bilirubin, which has protective anti-inflammatory effects, especially in vascular endothelial tissue [57]. Another anti-inflammatory action of HO-1 is through carbon monoxide production, which is a known cryoprotectant and anti-apoptotic factor in endothelial cells that have the potential to crosstalk with nitric oxide, a known vasodilator. Although this work serves as further evidence that HO-1 has important underlying anti-inflammatory and insulin-sensitizing mechanisms that may augment bilirubin’s therapeutic value, it is essential to note that the supporting evidence is not unequivocal. In contrast to these aforementioned findings, conflicting data suggest that HO-1 is implicated in driving inflammation and may even support insulin resistance in humans. Jais et al. demonstrated that HO-1 levels predict a strong positive prediction of metabolic disease in human subjects [58], while Ghio et al. reported HO-1 elevation due to cytokine stimulation in inflammatory disease [59]. Whether HO-1 is present in response to the inflammation or if it is the direct cause of inflammation is not completely clear.

Although HO-1’s direct role on insulin resistance and inflammation has not been fully elucidated, the influence of exercise on the HO-1 pathway may shed light on these equivocal data. Niess et al. showed that HO-1 expression in leukocytes increased significantly after sustained endurance exercise in marathon runners. The authors interpreted this to be due to the excessive amount of free radical production, although the mechanism that causes this upregulation of HO-1 in exercise is not completely clear [60]. However, it can be postulated that since exercise promotes ROS generation, it would induce nuclear factor (erythroid-derived 2)-like-2 (Nrf2) expression, which is a key transcription factor in inducing HO-1 [61,62,63]. A potential explanation for the upregulation of HO-1 may be that acute exercise can also propagate a transient pro-inflammatory state to increase levels of HO-1 via increased cytokine activity. Others have suggested that ROS, themselves, can induce and upregulate HO-1. Kurata et al. found that the HO gene was induced by 12-O-tetradecanoylphorbol 13-acetate response element (TRE) in the presence of hydrogen peroxide, a ROS [64]. These oxidative free radical levels vary based on habitually trained versus untrained subjects. The trained individuals had a much more robust adaptive antioxidant defense system and thus a lower level of ROS production [65]. HO-1 levels at rest are significantly reduced in trained subjects compared to untrained subjects [60]. This suggests an adaptive regulatory feedback mechanism to which, at rest, basal ROS are downregulated in trained individuals and hence, a lower HO-1 level. The prevailing hypothesis surrounding this observation is that HO-1 is upregulated to offer protection from the free radicals that are produced with exercise (Figure 3) [59]. 

### 3.2. The Emerging Role of Biliverdin Reductase in Immune Response

BVRA plays a vital role in macrophage polarization and as a target for regulating responses to bacterial lipopolysaccharides and complement activation products. BVRA is expressed in macrophages where it is tyrosine phosphorylated. Phosphorylated BVRA then binds to phosphatidylinositol 3-kinase (PI3K) at the p85α subunit to activate downstream signaling to Akt [66,67]. Macrophage classification occurs according to activation state and function. M-1 macrophages are classically activated macrophages that express cytokines such as TNFα and interleukin-17A. M-2 macrophages are alternatively activated macrophages that express anti-inflammatory cytokines such as interleukin-10 (IL-10) and transforming growth factor-beta (TGFβ). Overexpression of BVRA in macrophages elevates expression of M-2 macrophage markers, while knockdown of BVRA increases M-1 macrophage markers [68]. Renal ischemia-reperfusion injury increases the levels of BVRA positive macrophages increasing the levels of IL-10, helping in the reparative process [68]. The recruitment of macrophages is an influential process in the inflammatory response. Release of chemokines that act on specific receptors such as the complement activation fragment 5a receptor one (C5aR1) recruits macrophages to sites of tissue injury. Loss of macrophage BVRA results in greater levels of C5aR1 increasing inflammation [69]. These studies demonstrate the critical role of BVRA in both macrophage chemotaxis and polarization. Augmentation of macrophage BVRA levels may be an effective treatment to bolster anti-inflammatory pathways in a number of inflammatory diseases. How they might affect metabolic adaptations to exercise is yet to be determined. 

### 3.3. The Effect of Exercise on Bilirubin and Its Actions

**Figure 4:**
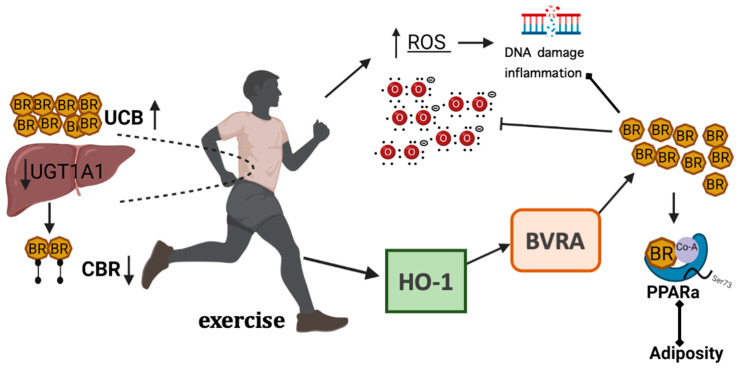
The heme oxygenase pathway signaling during exercise. Exercising (aerobic) raises plasma bilirubin levels by (1) suppression of the glucuronyl transferase enzyme UGT1A1 that conjugates bilirubin for removal from blood, and (2) activation of the heme oxygenase pathway (HO-1-BVRA-PPARα). The increased bilirubin combats reactive oxygen species (ROS) and ROS-induced inflammation and DNA damage. The bilirubin also activates the nuclear receptor transcription factor, PPARα, to reduce adiposity. Created with BioRender.com (accessed on 9 December 2021).

Given that HO-1 expression is directly influenced by exercise training (Figure 4), it is logical to assume that exercise increases plasma bilirubin levels. Hinds et al. recently conducted a study where rats genetically selected for high capacity running (HCR) and low capacity running (LCR) were used to identify the metabolic pathways in the liver altering plasma bilirubin levels through exercise [70]. The investigators observed that HCR rats had significantly greater plasma bilirubin and hepatic BVRA expression while having a reduced expression of the glucuronyl hepatic enzyme UGT1A1. Significant increases in PPARα-target genes were also observed in HCR rats compared to the LCR. For the first time, these results suggest hepatic mechanisms involved in bilirubin synthesis and metabolism that may explain the positive effects of exercise on plasma bilirubin and metabolic health.

There are a limited number of articles published on this topic in humans [71,72]. In a controlled study that examined different levels of training intensity, researchers found that the high-intensity training group (defined as 12 kilocalories per kilogram per week (KKW) energy expenditure) presented a significant increase in total serum bilirubin in comparison to the sedentary control group. Those who trained at moderate intensity levels (defined as 4 and 8 KKW) experienced no significant differences in serum bilirubin levels [71]. Priest et al. observed an increase in bilirubin in male runners after a 13-mile run along with an increase in alkaline phosphatase. Bile acids and bilirubin have been shown to be elevated in these runners [73]. In both studies, bilirubin levels seem to be elevated in response to high-volume, exhaustive forms of exercise with high energy expenditure. 

A subgroup analysis from Swift et al. revealed another interesting trend that showed those who were insulin resistant in the high-intensity exercise group had a significant increase in bilirubin compared to the insulin-sensitive group [71]. A more recent study confirmed these observations by examining why moderate-to-vigorous physical activity (MVPA) resulted in a significant increase in serum bilirubin in insulin-resistant subjects but not in insulin-sensitive subjects. The authors hypothesized that the observed increase in bilirubin in the insulin-resistant subjects could be due to a lower basal level of bilirubin, resulting in a more remarkable absolute change in bilirubin in response to MVPA [74]. The underlying rationale for this pattern of bilirubin change in response to different exercise volumes should be further explored to improve our understanding of the connection between insulin resistance and changing bilirubin levels. 

Several studies in athletes have also reported a strong correlation between elevated bilirubin and the degree of exercise intensity and an associated increase in erythrocyte hemolysis [71,75,76,77]. Witek et al. reported normal bilirubin reference ranges for 339 male and female Polish athletes [72]. While approximately 45% of the samples had bilirubin levels in the range of 7–14 μM, 12% of the athletes had 21–28 μM. Nineteen percent of the total bilirubin values exceeded the established normal limit of 21 μM. These elevated concentrations appeared to be related to changes caused by regular exercise and were not directly related to increased hemolysis. The authors suggested that other exercise-induced mechanisms seem to affect bilirubin concentrations, such as altered liver function and upregulation of bilirubin production (to serve as an antioxidant) in response to increased oxidative stress (ROS). A study of young Polish athletes (aged 18–40 years) reported that bilirubin levels increased in response to both a ketogenic diet and short-term, high-intensity exercise (CrossFit) [78]. Study subjects increased their bilirubin concentrations in both diet groups in response to exercise (Customary diet: 10 ± 5 to 19 ± 8; Ketogenic diet: 14 ± 0 to 20 ± 8 μM; *p* < 0.05). These studies bring to question if bilirubin levels are being controlled by exercise to correct metabolic imbalances, mitigate oxidative stress, and reduce inflammation.

### 3.4. The Hormonal Function of Bilirubin in Exercise and the Impact of PPAR Signaling

The PPARs are a subfamily of ligand-activated nuclear receptor transcription factors with three distinct isoforms: α, β/δ, γ [79]. These isoforms are found in different tissues, each with a predominant isoform. PPARα is expressed in hepatocytes, enterocytes, and vascular endothelium and works to improve mitochondrial efficacy in FA oxidation in these tissue types. PPARβ/δ are expressed more ubiquitously in the body but predominate in skeletal muscle and macrophages and are important in fatty acid oxidation and macrophage immunosuppression through the reduction in NF-κB inflammatory cytokines [80,81]. PPARγ is found mainly in white and brown fat adipocytes and enhances genes involved in the metabolism of glucose and adipocyte differentiation [82,83,84,85,86]. PPAR’s are activated in the presence of their specific corresponding natural or synthetic pharmacological ligands. All PPAR isoforms will activate in the presence of unsaturated fatty acid (PPAR pan agonist), which acts as a ligand to the PPAR isoforms [87]. It should be noted that all of the PPAR isoforms are considered to drive anti-inflammatory pathways. A hepatocyte-specific and adipocyte-specific knockout of PPARα in mice fed a high-fat diet showed greater fat content in each of the KOs, which both also exhibited significantly higher inflammation compared to control littermates [88,89]. Similarly, studies showing that overexpression of inflammatory meditator glucocorticoid receptor beta (GRβ) in the liver of C57/bl6 mice induced hepatic lipid accumulation in 5 days on a normal chow diet by suppression of hepatic PPARα [90]. 

**Figure 5:**
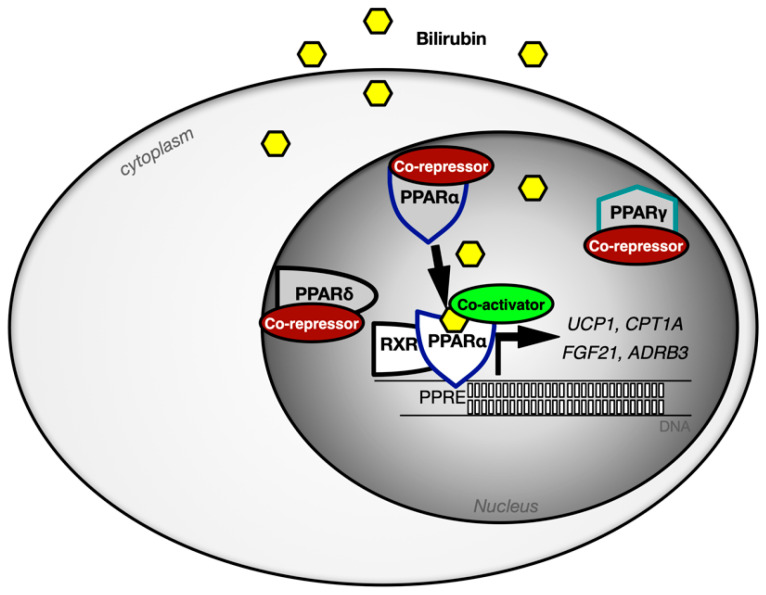
Selectivity of bilirubin for the PPAR isoforms and signaling mechanisms. The PPAR isoforms are bound by corepressors proteins until they are bound to the ligand, which induces a change from co-repressors to co-activators. Unconjugated bilirubin enters the cells and activates PPARα and not the PPARγ or PPARβ/δ isoforms. Bilirubin binding to PPARα induces a complex with RXR causing an exchange of corepressor proteins for co-activators. The bilirubin-induced PPARα-RXR complex controls specific genes for metabolic control of adiposity (*UCP1*, *CPT1A*, *FGF21*, *ADRB3*, and others), which might be based upon specific co-activators (PGC1α, NCOA1, NCOA2, MED1, etc.) bound in the complex.

We have shown that bilirubin (unconjugated form) binds directly to the PPARα nuclear receptor to induce transcription of genes (Figure 5) [91,92,93,94], which control adiposity and glucose sensitivity. Interestingly, competitive binding studies and transcriptional activity assays demonstrated that bilirubin’s binding to the PPARs is specific to only PPARα, and it has no actions or binding to PPARγ or PPARβ/δ [91,92]. In looking more specifically at ligands for PPARα, a synthetic ligand such as fenofibrate (fibrates) is widely used in the treatment of hypertriglyceridemia in order to reduce serum triglyceride levels. Through the binding and subsequent activation of the PPARα nuclear receptor, fenofibrate reduces plasma triglycerides and VLDL/LDL concentrations [95]. An increased expression of PPARα offers significant induction of β-oxidation [46,47,63,92,94] and myocardial ATP production, which are markers for myocardial viability [96,97]. It can also reduce the oxidative stress that occurs after a high-fat meal [95]. As mentioned above, unconjugated bilirubin has been demonstrated to act as a novel endocrine ligand that activates the transcriptional activity of PPARα by direct interaction, which changes coregulator proteins bound to the nuclear receptor to control gene activity [92]. PPARα activation by bilirubin in obese mice with glucose intolerance leads to a decrease in fasting blood glucose, as well increase in lean body mass and an increased presence of FGF21 (fibroblast growth factor 21) [42]. FGF21 can act as a metabolic regulator by rapid reduction in blood glucose and insulin levels in obese models [42,98] (readers are referred to another review discussing modulation of metabolism by FGF21 for more information [99]). The impact that bilirubin has on exercise via FGF21 is unknown. More studies are needed to elucidate the protective properties of bilirubin that occur via it driving the PPARα-FGF21 pathway that reduces adiposity and improves insulin sensitivity.

Exercise plays a role in the activation of the PPAR systems. Exercise increases the levels of AMP-activated kinase (AMPK), ERK1/2-MAPK, and PKC, which are kinases in the skeletal muscle involved in increasing the expression of downstream transcription factors. These kinases are found to increase the transactivation of PPARα and thus an increase in FA oxidation and glucose production, which can be used as fuel during exercise [100]. PPARα, in particular, has strong actions on improving the efficacy of FA oxidation in the liver and adipose tissues [88,89]. PPARα mRNA upregulates in bouts of exercise and in times of starvation in order to metabolize fat and use it for an effective energy source [101,102]. Acute exercise also provides increases in liver and serum FGF21, which provides systemic insulin sensitization [103]. PPARα expression is necessary for optimized endurance exercise. PPARα knockout models had significantly less tolerance to endurance exercise than the control. This lack of tolerance is due to a rapid depletion of hepatic glycogen [104]. We have shown that reducing PPARα activity in the liver leads to lower hepatic glycogen content [88,90], and activation by bilirubin increases it [47,70]. Similarly, hepatocyte-specific BVRA knockout animals on a high-fat diet had reduced bilirubin-PPARα activity and lower glycogen levels [50]. Endurance athletes were found to have a specific polymorphism that produces an increased binding capacity of PPARα in skeletal muscle and more type I slow-twitch fibers [105]. This suggests that PPARα may have critical roles in exercise and is necessary to perform enhanced endurance activity [106]. Similar to PPARα, PPARγ and PPARβ/δ mRNA is also elevated as a result of an aerobic exercise training program [107,108]. PPARβ/δ are the least studied of the isoforms. There is evidence to support PPARβ/δ’s ability to rectify metabolic disorders and enhance β-oxidation in muscle [109]. Many of its effects mimic the functionality of PPARα; however, the PPARβ/δ is more ubiquitously expressed than PPARα [110]. 

PPARγ is upregulated after sustained exercise programs and showed beneficial effects in skeletal muscle [111]. This skeletal muscle had signs of mitochondrial biogenesis and thus, improved aerobic respiration. The mitochondrial biogenesis is also seen in adipose tissue and is phenotypically evident by the increased conversion of white fat into brown fat in the presence of a highly induced PPARγ [112]. This exercise-induced PPARγ can provide antidiabetic effects through upregulation of monocyte PPARγ-control genes [111]. PPARγ is also in charge of controlling adipocyte differentiation [113,114]. In a PPARγ knockout model, severe lipoatrophy is observed, along with insulin resistance [115]. The PPARγ knockout mice have significantly decreased body mass; however, the liver showed a 1.5-fold increase in weight and increased lipid deposition in hepatic tissue. The increased lipid deposition in the liver is due to disrupted adipogenesis in white adipose tissue (WAT), causing increased plasma triglycerides that can deposit in the liver [116]. These PPAR systems have been correlated with decreased levels of atherosclerosis, insulin resistance, and inflammation in conjunction with metabolic syndrome and hypertriglyceridemia [114]. 

**Figure 6:**
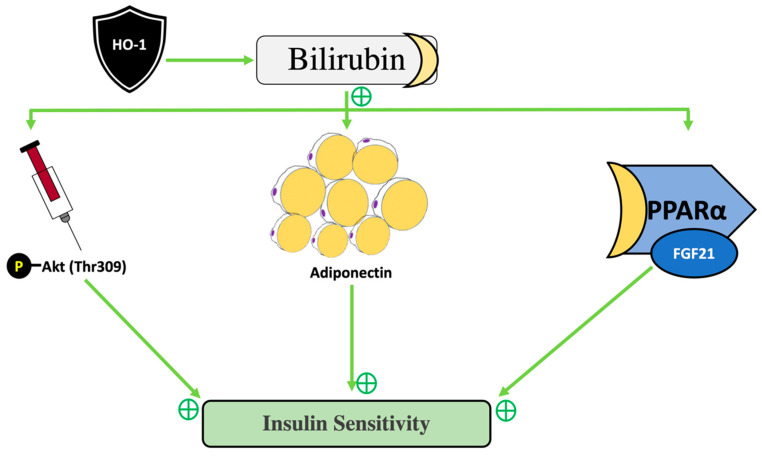
Model of how HO-1-bilirubin improves insulin sensitivity. Upregulation of HO-1-bilirubin is a multifactorial influencer of different metabolic processes such as induction phosphorylated Akt (Thr309), adiponectin production, and activation of PPARα-FGF21 pathways.

Another novel metabolic role designated to bilirubin is its natural ability to act as an insulin sensitizer [44,117]. PPARγ is elevated following bilirubin administration in mice with improved insulin sensitivity. This isomer of PPAR is implicated as a potent factor in adipocyte differentiation and adiponectin secretion [117]. Bilirubin administration has also improved obesity and hyperglycemia in rodent models. Bilirubin-treated obese mice increased phosphorylation of Akt (Thr309), an insulin-signaling molecule, in skeletal muscle and hepatocytes, indicating preservation of insulin sensitivity [44]. Bilirubin-treated mice also presented with greater adiponectin levels [117]. It should be noted that while bilirubin induced PPARγ expression in diabetic mice, it is not a ligand for this receptor as was previously demonstrated [91,92]. Because bilirubin levels rise with exercise more effectively in insulin-resistant subjects, there is therapeutic potential for bilirubin to control cholesterol metabolism and glucose tolerance in insulin-resistant patients. Therefore, exercise in pre-diabetic patients may offer metabolic benefits by raising HO-1, upregulating adiponectin and bilirubin levels, enhancing insulin signaling, activating PPARα pathways, and thus, decreasing insulin resistance (Figure 6).

## 4. The Signaling Mechanisms of Heme Oxygenase and Bilirubin in Metabolism

### 4.1. Generation and Catabolism of Bilirubin

Bilirubin is a tetrapyrrole compound formed from the catabolism of heme to biliverdin that is converted to bilirubin by biliverdin reductase (BVR) [17,41,42,118,119,120,121,122]. Tetrapyrroles are seen as an orange-yellow pigment, which may indicate underlying disease processes if extremely elevated (>150 μM) in the skin (jaundice) or the urine [41]. When erythrocytes (red blood cells) are lysed, the hemoglobin is broken down into heme and protoporphyrin. The heme is oxidatively cleaved by the enzyme heme oxygenase (HO), yielding biliverdin, iron, and carbon monoxide (CO) [41]. This biliverdin can be converted to bilirubin through the cytosolic enzyme biliverdin reductase [123,124,125]. The conversion to bilirubin has been empirically shown to produce potent antioxidant effects that can regulate cellular redox reactions, decrease ROS, and decrease the activity of NADPH oxidase [3]. Bilirubin circulates bound to water-soluble albumin, where it is transferred to the hepatocyte as unconjugated bilirubin. Then, bilirubin is conjugated by the UDP glucuronosyltransferase 1A1 (UGT1A1) enzyme [121,126]. Once conjugated, bilirubin is then metabolized by colonic bacterial proteases and is either reabsorbed into the hepatobiliary system as urobilinogen or excreted in the feces and urine as stercobilin or urobilin, respectively [122]. The bilirubin pathway (illustrated and described in more detail elsewhere [17,41,42,119,120,121,122,126]) is increased with exercise [70], and a better understanding of the pathway regulation may identify areas that alter the bilirubin half-life that might lead to pathological consequences.

### 4.2. Biliverdin Reductase and Metabolism

While there are limited studies showing that BVRA is regulated by exercise [70], there have been supporting studies showing a role for the enzyme in metabolism [50,51,124,125,127]. Adipocyte-specific deletion of BVRA results in adipocyte hypertrophy and increased inflammation while decreasing mitochondrial number and markers of adipocyte browning such as PPARα and β3 adrenergic receptor (*Adrb3*) [127]. The loss of adipocyte BVRA also decreases insulin signaling in white adipose tissue contributing to increased fasting hyperglycemia in knockout mice [127]. These results agree with the finding from obese human patients who exhibit lower levels of BVRA, increased levels of inflammation, and increased adipocyte size [128]. CRISPR knockout of BVRA in hepatocytes and kidney proximal tubules cells induces oxidative stress and lipid accumulation [124,125]. Similarly, mice with a global knockout of BVRA have increased oxidative stress [41]. Deficiencies in BVRA also correlate with brain insulin resistance in Alzheimer’s disease patients [129,130]. Administration of BVRA peptides improved intranasal insulin treatment in a mouse model of Alzheimer’s disease, suggesting a potential therapeutic role for targeting BVRA for treatment [131]. 

BVRA also plays an essential role in the development of metabolic diseases associated with obesity, like NAFLD. Hepatocyte-specific BVRA knockout mice develop more severe dietary-induced NAFLD as compared to wild-type littermates [50]. The loss of hepatocyte BVRA increases activation of glycogen synthase kinase 3beta (GSK3β) via decreased levels of serine 9 (Ser9) phosphorylation which in turn increases serine 73 (Ser73) phosphorylation of PPARα, increasing protein turnover and decreasing its transcriptional activity [50]. Interestingly, reduced adipocyte levels in BVRA in obese human patients resulted in significantly more hepatic steatosis and NAFLD [128]. These results suggest that BVRA can have both direct and indirect effects to contribute to hepatic steatosis and the development of NAFLD. More studies are needed to determine factors that regulate BVRA expression and how these are affected by exercise. 

### 4.3. Bilirubin and Metabolic Dysfunction

Bilirubin was once believed to act only as a toxic bile substance and end-product. However, more recent studies have uncovered potential metabolic benefits of greater yet subclinical bilirubin concentrations. These include its role in ROS scavenging, anti-inflammatory properties, and reduction in adipocyte size from increased fat oxidation [94,132,133]. While marked extreme hyperbilirubinemia (>150 μM) can be a sign of a more ominous clinical diagnosis, raised basal concentrations are also associated with protecting metabolic function (25–50 μM as discussed in [41]). The metabolic syndrome [134] is associated with increased insulin resistance and oxidative stress, which can also lend significant inflammatory and cardiovascular risk factors. Increased serum bilirubin concentration acts as a protective factor against the development of MetS. Subjects with increased basal bilirubin levels have a lower odds ratio to develop MetS [134]. It is thought this observation is due to the antioxidant, anti-inflammatory, and hormonal properties of bilirubin (discussed in more detail above). Conversely, in subjects diagnosed with metabolic syndrome, serum bilirubin is typically reduced (<10 μM, discussed further in [41]) [135]. Thus, the clinical assessment of serum bilirubin may have some future utility as a screening or prediction tool for those with high risk for metabolic dysfunction. In support of this, coronary artery disease severity was recently predicted with an odds ratio of 0.155 (95% confidence interval), revealing an inverse relationship between bilirubin and CAD severity [136]. NAFLD was also predicted in patients with an odds ratio of 0.88 (95% confidence interval), showing a strong inverse relationship between serum bilirubin and NAFLD [137]. A study of obese children showed that those with NAFLD had the lowest serum bilirubin [133]. Low bilirubin has also been associated with a greater risk of cerebral deep white matter lesions in healthy subjects [138], suggesting that low levels may impair cognitive function or lead to stroke [139,140,141]. These studies might suggest that increasing bilirubin levels could be therapeutic for improving metabolic dysfunction and reducing stroke risk. Factors that induce heme oxygenase production of bilirubin, such as nutraceuticals, may have several benefits [63]. These studies highlight a potential protective effect of bilirubin against metabolic disease and should be examined further to elucidate more of its positive benefits.

## 5. Strategies to Improve Metabolic Outcomes through Nutraceuticals

### 5.1. The Influence of Diet on Antioxidants

Fresh fruit and vegetable intake represent the largest source of dietary antioxidants that are essential in maintaining health [142]. A great deal of research has evaluated fruit and vegetable intake as a means to counter the inflammation that has been attributed to nearly all chronic diseases of modern society, with many studies demonstrating a strong inverse correlation between fruit and vegetable intake and inflammatory markers [143,144]. Much of the research concerning dietary antioxidants and inflammation has centered on the Mediterranean diet due to its emphasis on fresh fruit and vegetable intake. Both cross-sectional and longitudinal trials have demonstrated a substantial lowering effect for the Mediterranean diet on a wide variety of inflammatory markers, including IL-6, IL-7, IL-19, CRP, and TNFα [145,146,147]. This has prompted the use of the Mediterranean diet in hopes of managing metabolic and vascular diseases, endocrine disorders, and some cancers [148,149,150]. Elucidation of specific antioxidants, and the benefits of dietary supplementation, have been a focus for many current research studies, giving rise to various nutraceuticals. Below, we describe the effects of such nutraceuticals and their beneficial actions on the HO-1 pathway and inflammation. 

### 5.2. The Benefits of Moderately Raising Plasma Bilirubin

Natural substances that raise plasma bilirubin have been of interest for reducing adiposity [63]. One herbal method that is gaining interest in elevating plasma bilirubin is the use of the milk thistle plant (*Silybum marianum*) [63]. The plant contains a mix of polyphenols such as p-coumaric, vanillic acid, silybin, and α-tocopherol [151,152]. The primary compound in milk thistle that is considered the active component that increases plasma bilirubin is the silymarin flavonoids that suppress hepatic UGT1A1 [153]. Silymarin may protect against liver injury and hepatic fat accumulation [154,155,156]. However, how milk thistle or silymarin might function combined with exercise in reducing adiposity is unknown. 

Bilirubin is a potent endogenous antioxidant that the body uses to support oxidative balance [17,41,42,119,120,121,122]. Plasma bilirubin has been empirically correlated with decreased risk for oxidative disorders such as coronary artery disease (CAD) [42,119]. The theory of action stemmed from individuals with Gilbert Syndrome, who have a mutation in the UGT1A1 gene, which causes defective processing of bilirubin [47,126]. Hence, lower hepatic UGT1A1 causes higher plasma unconjugated bilirubin [47]. Individuals with Gilbert Syndrome were found to have decreased incidence of CAD [157]. Previous studies postulate that unconjugated bilirubin is fluxing back into cells and acting as a scavenging agent of oxidative radicals. However, researchers have also hypothesized that elevated serum bilirubin acts as a marker that could predict greater expression or inducibility of intracellular HO-1, which will increase the intracellular concentration of bilirubin [132]. Using HPLC-TLS, these researchers detected bilirubin levels within vascular endothelial cells. They also showed that bilirubin within these vascular endothelial cells could effectively modulate HO-1 upregulation [158]. These findings suggest strong potential for developing pharmacotherapeutics that can target and upregulate this intrinsic antioxidant system within the vascular endothelium through the induction of HO-1 and help prevent or follow the progression of cardiovascular disease. This has the potential to lend more focused antioxidant and anti-inflammatory therapeutic approaches.

### 5.3. Vitamin D Repletion

Vitamin D is an important secosteroid in understanding metabolic disease [159]. Vitamin D deficiency (defined as a 25(OH)D level less than 20 ng/dL) is common and is associated with decreased muscle endurance, function, and strength [160,161,162,163,164,165,166,167]. Vitamin D deficiency is connected to muscle metabolic perturbations, including insulin resistance [168,169,170], and is linked to mitochondrial dysfunction [171] in both young and aged adults. Vitamin D deficiency is highly prevalent in obesity without vitamin D supplementation [172,173]. Obese adults are commonly prescribed a high-dose vitamin D repletion protocol to combat vitamin D deficiency and obesity-associated vitamin D resistance. Aggressive vitamin D repletion to correct the deficiency is linked to improved muscle mitochondrial function [171,174]. Increasing vitamin D status is consistently associated with skeletal muscle lipid deposition and distribution [175,176,177,178]. There is also evidence that vitamin D may improve hepatic steatosis with just 4-weeks of supplementation [179]. 

Calcitriol, the active form of vitamin D [1,25(OH)_2_D_3_], is the only form that can bind to the Vitamin D receptor (VDR). The VDR is a nuclear receptor transcription factor that controls gene expression changes that improve mitochondrial function in myotubes [180], insulin sensitivity, and myocellular lipid partitioning in high fat-treated SkM cells [181]. In humans, we found that vitamin D combined with aerobic exercise potentiated the metabolic benefit of training by producing the most intramyocellular lipid (IMCL) loss and increasing skeletal muscle tissue-level VO_2_ in older adults at risk for metabolic dysfunction [182]. These benefits were greater than when providing vitamin D repletion or exercising independently. These observations are consistent with reports that vitamin D coupled with exercise has positively affected muscle mitochondrial function [171,174]. In addition, VDR expression in SkM was increased by exercise [183]. Vitamin D supplementation has been associated with muscle regeneration and repair [123,145,146], suggesting an additive effect when combined with vitamin D repletion. 

In addition to these findings, vitamin D has been described to have anti-inflammatory effects and is linked to insulin sensitivity and immuno-modulation [184,185,186]. Recent work has also highlighted a novel role of vitamin D in upregulating HO-1 expression in intestinal cells and reduced expression of macrophage HO-1 with an associated reduction in conjugated bilirubin [187]. Vitamin D has been shown to block the activation of M-1 macrophages, increase activation of M-2 macrophages, and impair monocyte/macrophage recruitment [187]. Collectively, these data suggest that vitamin D may ameliorate metabolic dysfunction by altering lipid availability for oxidation in response to exercise training and may help regulate inflammatory pathways. These observations require further exploration in obesity-inflammation studies.

Along with evidence that vitamin D repletion augments oxidative metabolism [171,174], we show in a muscle cell line that active calcitriol treatment altered total lipid, lipid species content, and increased gene expression of PLIN2, a lipid coatomer protein that facilitates IMCL availability for β-oxidation [188,189]. PLIN2-containing lipid droplets are also preferentially used during moderate-intensity exercise [190], suggesting that increased PLIN2 expression may increase lipolytic potential. In vitro findings from our group [180,181] indicate increased PLIN2-associated lipid accumulation and lipolysis after calcitriol treatment. These changes suggest an increased lipid flux—defined here as the rate at which lipids pass through SkM via IMCL accumulation and oxidation—and, by association, a decrease in lipid-mediated pathologies [191]. These cell culture results suggest that vitamin D is involved in muscle lipid packaging, partitioning, and mitochondrial lipid oxidation. Together with data showing that exercise improves muscle sensitivity to vitamin D storage and retention [192], evidence of muscle adaptations to the combination of vitamin D supplementation with exercise are tightly connected with improved mitochondrial function and may serve an integral role in delaying stagnant ectopic fat infiltration and metabolic dysfunction.

### 5.4. Nitrate from Foods and Dietary Supplements

Dietary nitrate is predominately found in green leafy vegetables and concentrated food sources (e.g., beetroot juice) and dietary supplements. Physically active individuals commonly use this EE to increase plasma nitrate concentrations and subsequently increase nitric oxide availability [193]. Increasing nitric oxide via the nitrate-nitrite-NO signaling pathway (with supplemental dietary nitrate) has been shown to decrease NADPH oxidase-derived oxidative stress via HO-1 induction and reduce p47phox expression [194]. Metabolically, nitrate has also been shown to reduce the oxygen cost of exercise [195] and improve exercise tolerance, economy, and performance. These benefits may also extend to those newly committed to exercise to lose fat and improve metabolic function. In addition to these observations, it seems reasonable that dietary nitrate may also work alongside exercise to preserve endothelial function [196]. Basaqr et al. recently found that four weeks of concentrated beetroot juice combined with vitamin C improved endothelial function and the lipid profile of overweight subjects with evidence of endothelial dysfunction [197]. The exact mechanism of action to explain these findings is unknown but is partially explained by the combined antioxidant effects of vitamin C and concentrated dietary nitrate supplements to decrease oxidative stress [198,199,200,201]. Improvements in blood lipids from others suggest that dietary nitrate supplementation with the addition of vitamin C (or other nutraceutical antioxidants) may be a valuable dietary approach alongside exercise to improve metabolic and cardiovascular health [60,61]. Future studies could determine how these impact exercise, inflammation, and metabolic outcomes. 

### 5.5. Vitamin E Supplementation

Vitamin E (α/γ-tocopherol) is one of the most important dietary antioxidants that play a critical role as a radical savaging agent and mechanistic inducer [202]. Vitamin E acts as a potent antioxidant to neutralize free radicals and superoxide by using its free hydroxyl group to accept unpaired electrons [203]. Furthermore, unlike other dietary antioxidants (e.g., vitamin C, carotenoids, etc.), Vitamin E is uniquely connected with exercise-induced oxidative stress and insulin sensitivity. The regulation and distribution of Vitamin E are controlled by alpha-tocopherol transfer protein (α-TTP) in the liver. α-TTP secretes Vitamin E from the liver by releasing α-tocopherol into the circulation. However, this mechanism is still not clearly understood [204]. Data suggests that α-TTP is lower when the α-tocopherol levels are low and subjects with an α-TTP gene (TTPA) knockout presented with symptoms of vitamin E serum deficiency [202]. The α-TTP is also known to be induced by hypoxic states and stress-induced free radical production [205]. The administration of vitamin E in hypoxic states (similar to hypoxia observed with high-intensity exercise) has mitigated ROS-related biochemical changes in many tissues by preventing increases in malondialdehyde and myeloperoxidase and protecting against lipid peroxidation [206]. This hypoxia-based regulative mechanism has the potential to be evident during times of exercise; however, this hypothesis has yet to be tested. The rationale behind this hypothesis is that during exercise, hypoxia-induction of α-TTP will help increase serum α-tocopherol and protect the cells from free-radical damage during exercise-induced oxidative stress. This tocopherol can also provide non -antioxidant functions and induce mRNA levels of transcription factors, PPARγ, and the hormone, adiponectin [207,208]. Adiponectin and PPARγ are activated by vitamin E and are known to improve insulin sensitivity in diabetes. The vitamin E-induction of PPARγ is not through direct binding but through the increase in 15d-PGJ2, a commonly described ligand of PPARγ [208] that is also known to induce HO-1 through p38 MAP kinase and the Nrf-2 pathway [209]. This induction may further increase exercise-induced HO-1 activation and influence the BVRA-bilirubin-PPARαaxis. Both Vitamin E and PPARs are valuable targets in hepatic protection in non-alcoholic hepatic steatosis and fibrosis [210]. 

## 6. Conclusions

Exercise has clear benefits in reducing adiposity and inflammation while improving insulin sensitivity. A deeper understanding of the mechanisms of how exercise functions to improve these beneficial actions is needed. While bilirubin was once thought to be a harmful bile substance, current research argues otherwise and that slightly elevated levels have numerous health benefits against metabolic dysfunction. Studies on how exercise influences factors such as heme oxygenase, BVRA, and UGT1A1 that control bilirubin’s turnover (half-life) are needed. Furthermore, nutraceuticals that activate and control these pathways might be beneficial in improving weight-loss regimens. Investigations in these areas might also benefit patients with inflammatory disorders as increasing plasma bilirubin has anti-inflammatory properties that probably originate from its antioxidant and hormone (as a ligand for PPARα) properties. Future work determining the interplay of exercise and nutraceuticals has many health benefits to help a broad spectrum of diseases.
